# Ligation Bias in Illumina Next-Generation DNA Libraries: Implications for Sequencing Ancient Genomes

**DOI:** 10.1371/journal.pone.0078575

**Published:** 2013-10-29

**Authors:** Andaine Seguin-Orlando, Mikkel Schubert, Joel Clary, Julia Stagegaard, Maria T. Alberdi, José Luis Prado, Alfredo Prieto, Eske Willerslev, Ludovic Orlando

**Affiliations:** 1 Centre for GeoGenetics, Natural History Museum of Denmark, University of Copenhagen, Copenhagen, Denmark; 2 Centre de Conservation et d’Étude des Collections, Musée des Confluences, Lyon, France; 3 Ree Park, Ebeltoft Safari, Ebeltoft, Denmark; 4 Departamento de Paleobiología, Museo Nacional de Ciencias Naturales, Consejo Superior de Investigaciones Cientificas, Madrid, Spain; 5 Investigaciones Arqueológicas y Paleontológicas del Cuaternario Pampeano, Consejo Nacional de Investigaciones Científicas y Técnicas - Universidad Nacional del Centro de la provincia de Buenos Aires, Olavarría, Argentina; 6 Instituto de la Patagonia, Universidad de Magallanes, Punta Arenas, Chile; Programa de Doctorado, Universitat Autònoma de Barcelona, Barcelona, Spain; Deutsches Krebsforschungszentrum, Germany

## Abstract

Ancient DNA extracts consist of a mixture of endogenous molecules and contaminant DNA templates, often originating from environmental microbes. These two populations of templates exhibit different chemical characteristics, with the former showing depurination and cytosine deamination by-products, resulting from *post-mortem* DNA damage. Such chemical modifications can interfere with the molecular tools used for building second-generation DNA libraries, and limit our ability to fully characterize the true complexity of ancient DNA extracts. In this study, we first use fresh DNA extracts to demonstrate that library preparation based on adapter ligation at AT-overhangs are biased against DNA templates starting with thymine residues, contrarily to blunt-end adapter ligation. We observe the same bias on fresh DNA extracts sheared on Bioruptor, Covaris and nebulizers. This contradicts previous reports suggesting that this bias could originate from the methods used for shearing DNA. This also suggests that AT-overhang adapter ligation efficiency is affected in a sequence-dependent manner and results in an uneven representation of different genomic contexts. We then show how this bias could affect the base composition of ancient DNA libraries prepared following AT-overhang ligation, mainly by limiting the ability to ligate DNA templates starting with thymines and therefore deaminated cytosines. This results in particular nucleotide misincorporation damage patterns, deviating from the signature generally expected for authenticating ancient sequence data. Consequently, we show that models adequate for estimating *post-mortem* DNA damage levels must be robust to the molecular tools used for building ancient DNA libraries.

## Introduction

The preservation of DNA in fossil specimens has opened new perspectives in evolutionary biology, providing access to genetic information from past individuals [1], [2] and extinct species [3], [4]. Ancient DNA research has long been limited to the retrieval of short sequence information from organelle genomes [5] but recent developments in next-generation sequencing (NGS) technologies have circumvented intrinsic methodological limitations, providing megabase-scale datasets [6]–[8] to complete ancient genomes [1]–[4], [9]–[11] ranging from a hundred of years [2] to several hundreds of thousands of years [12].

Most ancient DNA extracts consist of a mixture of DNA templates originating from the fossil specimens themselves as well as environmental microbes that colonize fossils after death [13]. Microbial contamination typically outnumbers endogenous DNA by one or two orders of magnitude [3], [14], except in rare situations, such as well-preserved keratinous tissues, in which the endogenous DNA dominates [1], [2]. The massive throughput of NGS-based approaches, enabling the characterization of millions to billions of sequencing reads in no more than a few days [15], has been essential for enabling the identification of the endogenous minority of reads. Additionally, base composition patterns have been critical for distinguishing among truly ancient sequence data and contamination [14], [16]. One such procedure is based on the signature of the most prominent form of *post-mortem* DNA damage, namely cytosine deamination, which transforms native cytosines into uracils [17], [18]. Replication over uracil residues during DNA library preparation and amplification generates spurious C→T and G→A misincorporations, especially at sequencing termini where deamination rates are increased by orders of magnitude due to the presence of single-stranded overhangs in ancient DNA fragments [16].

Following the most popular library preparation method for ancient DNA that is based on the ligation of blunt-ended inserts [11], [19]–[23], such patterns can be recognized when aligning ancient reads against a modern reference genome by decreasing rates in C→T mismatches from the start of sequences, and a complementary increase in G→A rates towards the ends of sequences [24]. Other patterns, such as the presence of a greater-than-expected purine frequency at the genomic position preceding the start of sequences, have also been proposed as the hallmark of depurination-driven fragmentation of genuine ancient DNA templates after death [14], [20]. Depurination and cytosine deamination have recently been confirmed as driving-forces of *post-mortem* DNA damage using procedures that target single-stranded templates coupled with Illumina [4] and true Single Molecule DNA Sequencing [21], [25]. Although specific pre-extraction procedures, such as bleach treatment, could damage contaminant DNA and result in misincorporation and fragmentation patterns similar to those observed with ancient templates [26], such patterns are generally considered as essential for authenticating ancient DNA data [14], [20] and computational packages have been released for assessing their presence in a user-friendly manner [24], [27].

Experimental data have also revealed that different misincorporation and fragmentation patterns will be generated depending on the molecular tools used before generating sequence data. In particular, specific enzymatic treatments of DNA extracts before library preparation could result in the formation of single strand DNA breaks at sites containing deaminated cytosines [28], removing most of the misincorporation pattern. Similarly, once libraries have been prepared, PCR amplification with *Taq* polymerases that cannot bypass uracils will remove the expected C→T declines at start positions within sequences [1], [24]. One such DNA polymerase is *Phusion*, that is part of the standard Illumina library preparation protocol. The library preparation procedure itself has also been shown to generate different misincorporation patterns. For instance, in a recent library building procedure targeting single-stranded DNA templates, the misincorporation rate of C→T instead of G→A is inflated at sequence ends, as a result of the presence of 3′-overhangs [4].

How much misincorporation and fragmentation patterns might be modified by other DNA library preparation procedures has yet to be investigated. Consequently, it remains unknown which patterns could serve as authentication criteria for another popular DNA library preparation, one that is based on ligation at AT-overhangs [29]. In this procedure, after a first step of end-repair, 3′-dA tails are added to DNA templates following an elongation step with an enzyme showing 5′- to 3′- DNA polymerase activity. Adapters with 5′-dT overhangs are then ligated to DNA inserts before libraries can be PCR amplified and sequenced. This procedure has been developed as an alternative to the blunt-end ligation approach as the presence of 3′-dA and 5′-dT overhangs precludes adapter self-ligation and has been used in a range of ancient DNA studies [1], [8], [30], [31].

In this study, we characterize the base composition bias related to blunt-end *versus* AT-overhang DNA library preparation methods using fresh DNA extracts. We then address whether such bias is sensitive to different DNA fragmentation methods. Finally, we show how AT-overhang DNA library preparation methods modify the canonical DNA damage patterns expected for authenticating ancient DNA data.

## Materials and Methods

### Samples

Four different samples were included in this study. Two samples consisted of modern fresh material, namely onager (a species of modern equid, *Equus hemonius onager*) blood, and the bacteria *Escherichia coli*. Two ancient samples were also analyzed, one consisting of a quagga museum specimen [31] (*Equus quagga quagga*), and the other corresponding to a Late Pleistocene *Hippidion* bone (*Hippidion saldiasi*). The two ancient samples were processed at the Centre for GeoGenetics in state-of-the-art lab facilities dedicated to the analysis of fossil material. These facilities are located in buildings physically separated from post-PCR laboratories where the analysis of modern samples is performed.

### Ethics Statement

Onager blood samples (for a total of 50 ml stored in 5 EDTA-coated tubes) were taken by Ebeltoft Safari - Ree Park veterinary staff, when the animal was under anesthesia at the occasion of a medical hoof treatment. Blood samples were primarily taken for the Park blood bank, as a tool for disease investigation and surveillance, and at the same occasion extra samples were collected for the study.

The *Hippidion saldiasi* sample was excavated in the Ultima Esperanza cave (Milodon cave, dated 11,480 ± 60 years Before Present), Ultima Esperanza province, Magallanes, South Chile. It was stored at the Museo Argentino de Ciencas Naturales « Bernardino Rivadavia » in Buenos Aires, Argentina, under the specimen number MACN-5868.

The quagga sample was sent to us by the Musée des Confluences, Lyon, France, where it is registered under the specimen number 40000218.

### Modern DNA extraction and fragmentation

A total of 6 ml of frozen onager blood was extracted using the QIAamp DNA Blood Midi Kit (QIAGEN, reference nb. 51183), following the manufacturer’s recommendations, except that Proteinase K incubation was performed for 30 min at 70°C and that elutions were performed twice, in 300 µl AE buffer and 150 µl AE buffer, respectively. Three extractions of 2 ml of blood each were done in parallel and final eluates were pooled together.

Fresh bacterial DNA was extracted from cell cultures, following an incubation for 16 h of a suspension of 1 µl One Shot TOP10 Chemically Competent *E. coli* (Invitrogen) in 10 mL LB at 37°C, with gentle shaking at 200 rpm. Four DNA extractions were done using 1 ml of bacterial culture and the DNeasy Blood and Tissue Kit (QIAGEN, reference nb. 69506), following manufacturer’s instructions and 1 h incubation in the lysis buffer. Extractions were eluted in 200 µl AE buffer each and pooled together. All extractions included appropriate controls.

We measured the concentration of all fresh DNA extracts using a Qubit dsDNA HS assay (Invitrogen). Aliquots of 500 ng of *E. coli* and *E. h. onager* extracts were subjected to three parallel fragmentation procedures, using either the Bioruptor NGS (Diagenode), the Covaris E210 sonicator (Covaris) or a Nebulizer Kit (part of the Paired-End DNA Sample Preparation Kit, Illumina). Briefly, Bioruptor fragmentation was performed with DNA extracts diluted in TE buffer to a final volume of 100 µl, and using 20 cycles of 30’’/30’’ (ON/OFF cycles). For Covaris fragmentation, DNA extracts were diluted up to 130 µl in TE buffer in microTUBE AFA Fiber with Crimp-Cap (Covaris), and sonicated for 480 seconds using a duty cycle of 10%, an intensity of 5, and 200 cycles per burst. For the nebulization procedure, DNA extracts were diluted up to 50 µl in TE buffer, and added to 700 µl nebulization buffer (53% Glycerol, 37 mM Tris-HCL, 5.5 mM EDTA) in Illumina supplied nebulizers, and subjected to 2.4 bar compressed nitrogen for 8 min. Nebulized products were purified on a Qiaquick column following manufacturer’s instructions and eluted in 50 µl EB. All Qiagen column purification steps in this study were done using a final elution step at 37°C for 15 min.

Fragmented DNA samples were run on a 2% agarose gel, along with a 50 bp ladder (GeneRuler, Thermoscientific), size selected between 125–175 bp and purified using the QIAquick Gel Extraction Kit (QIAGEN, reference nb. 28704). Size distribution and concentration were checked on a 2100 Bioanalyzer (Agilent) High Sensitivity DNA Assay. Aliquots of 3 ng of fragmented and size-selected DNA were further used for preparing Illumina DNA libraries.

### Modern DNA library preparation

Two different types of Illumina DNA libraries were built. The first library type was based on blunt-ended adapter ligation and corresponds to the most commonly used library building method in ancient DNA research [19]. The second library type was based on AT-overhang adapter ligation and corresponds to standard Illumina library building procedures.

Blunt-end (BE) libraries were built following Kircher and Meyer [19] with 0.6 µM as a final concentration of Illumina multiplex adapters (5′-ACA CTC TTT CCC TAC ACG ACG CTC TTC CGA TCT and 5′-AGA TCG GAA GAG C for one adapter, 5′-GTG ACT GGA GTT CAG ACG TGT GCT CTT CCG ATC T and 5′-AGA TCG GAA GAG C for the other). All SPRI purification steps were replaced by spin column purifications using the MinElute PCR Purification Kit (QIAGEN). Libraries were all purified on a MinElute column and eluted in 30 µl EB buffer following 37°C incubation for 15 min. A-tailed (AT) libraries were built with NEBNext Quick DNA Library Prep Master Mix Set for 454 (New England BioLabs, reference nb. E6090), without the small fragment removal step, using either 0.012 µM (low concentration) or 0.6 µM (standard concentration) of Illumina inPE adapter (5′ P-GAT CGG AAG AGC ACA CGT CT and 5′-ACA CTC TTT CCC TAC ACG ACG CTC TTC CGA TCT).

DNA libraries were amplified using Micellula DNA Emulsion and Purification Kit (Roboklon, reference nb. E3600) following manufacturer’s instructions to perform emulsion PCR. The water phase consisted in a 50 µl volume PCR mix, using 5 µl of DNA library, 1 µM of Primer inPE1.0 (5′-AAT GAT ACG GCG ACC ACC GAG ATC TAC ACT CTT TCC CTA CAC GAC GCT CTT CCG ATC T), 20 nM of primer inPE2.0 (5′-GTG ACT GGA GTT CAG ACG TGT GCT CTT CCG ATC T), 1 µM of an Illumina multiplex primer (5′-CAA GCA GAA GAC GGC ATA CGA GAT NNN NNN GTG ACT GGA GTT C, where the N stretch corresponds to a 6 nucleotides index tag), 1 unit *Phusion* High-Fidelity DNA Polymerase (New England BioLabs, reference nb. M0530) and 200 µM of each dNTP (Invitrogen), in a 1X Detergent-free *Phusion* HF Buffer (New England BioLabs, reference nb. B0520). PCR cycling conditions consisted of initial denaturation for 30 sec at 98°C, followed by 15 cycles of 10 sec denaturation at 98°C, 30 sec annealing at 65°C and 30 sec elongation at 72°C. Lastly, there was a final 5 min elongation step at 72°C. After emulsion breaking using Isobutanol (Merck Millipore), PCR products were purified on Roboklon provided columns and eluted in 50 µl EB following 15 min incubation at 37°C. Other amplification conditions in absence of emulsion were also tested and did not affect our main results (Material and Methods S1).

### Ancient DNA extraction

The quagga specimen was extracted as described in [31]. The *Hippidion* bone sample was extracted using the silica-based DNA extraction method described in [32], with slight modifications [33]. Overall, a total of 151 mg of bone powder was digested overnight at 37°C in 5 ml of 0.5 M EDTA, 1 mg/mL Proteinase K, 0.5% *N*-lauryl-sarcosyl digestion buffer. Following centrifugation for 2 min at 2,000 rpm, the supernatant was recovered and further incubated for 3 hours at room temperature with 100 µl of resuspended silica pellets and 20 ml binding buffer (5 M GuSCN, 20 mM EDTA, 25 mM NaCl, 50 mM Tris and 1.3% Triton X-100). We adjusted pH at 4.0–5.0 with 37% HCl before starting the incubation. Silica pellets were recovered following incubation using 2 min centrifugation at 2,000 rpm and washed twice with 1 ml cold 80%-ethanol solution freshly prepared. Finally, DNA was eluted using 300 µl of TE buffer (10 mM Tris, 1 mM EDTA) following 15 min of incubation at 37°C.

### Ancient DNA library preparation

Each library was built using an aliquot of 16 µl of DNA extract and following two different protocols: the first using A-tailed adapters (inPE adapters), and the second based on blunt-ended adapter ligation [19]. DNA libraries were prepared following the same procedure as for modern DNA extracts, with minor modifications. Each library building reaction was purified on a MinElute column (QIAGEN) and eluted in 32 µl EB buffer following 15 min incubation at 37°C.

For BE libraries, we used the NEBNext DNA Library Prep Master Mix Set for 454 (New England BioLabs, reference nb. E6070) with 10 pmol Illumina multiplex adapter [19] following the manufacturer’s instructions without ssDNA Isolation Module. After ligation, the reaction was cleaned up on a MinElute column (QIAGEN) and eluted in 42 µl EB following 15 min incubation at 37°C. We added 5 µl Adapter Fill-in Reaction Buffer and 3 µl *Bst* DNA polymerase, Large Fragment to the full eluate volume and incubated the reaction at 37°C for 20 min. For AT libraries, we used the NEBNext Quick DNA Library Prep Master Mix Set for 454 (New England BioLabs, ref : E6090), without the small fragment removal step, and with 10 pmol Illumina inPE adapter.

The libraries were first amplified in a 50 µl volume reaction using 5 µl of DNA library, and 5 units *Taq* Gold (Life Technologies), 1X Gold Buffer, 4 mM MgCl2, 1 mg/ml BSA, 62.5 µM of each dNTP, 0.5 µM of Primer inPE1.0, 10 nM of Primer inPE2.0 and 0.5 µM of an Illumina multiplex primer, as described with the modern samples. PCR cycling conditions consisted of initial denaturation for 10 min at 92°C, followed by 12 cycles of 30 sec denaturation at 92°C, 30 sec annealing at 65°C and 3 min elongation at 72°C. Lastly, there was a final 7 min elongation step at 72°C. PCR products were purified on a MinElute column and eluted in 20 µL EB following 15 min incubation at 37°C. A second round of PCR amplification was then performed by splitting the purified product of the first PCR amplification into four reactions of 50 µl each using similar conditions but without inPE2.0 primer and only using 10 cycles. The four reactions were pooled and purified on a single MinElute column, eluted in 20 µl EB following 15 min incubation at 37°C. Other amplification conditions with shorter elongation steps of 40 sec were also tested and did not affect our main results (Material and Methods S2).

### DNA Sequencing

Amplified libraries were quantified using the 2100 Bioanalyzer (Agilent) High-Sensitivity DNA Assay. In case a high amount of adapter dimer was present, size selection was performed using either an E-Gel SizeSelect 2% Agarose electrophoresis (Invitrogen), or a LabChip XT 750 DNA Assay (Caliper) following the manufacturer’s instructions. Libraries were pooled with other indexed DNA libraries and Paired-End sequenced (2×100) over 4 lanes on Illumina Hiseq 2000 platforms at the Danish National High-Throughput DNA Sequencing Centre (Table 1), except for one that was pooled with other unrelated DNA libraries and sequenced as Single-End (100 cycles). All sequence data generated in this study have been submitted to Sequence Read Archive under accession number (SRA099068).

**Table 1:** Sequence data.

Species	Library	Adapter	Shearing	#Raw	#Collapsed	#Hits	Clonality	Endogenous
*E. coli*	BE	S	C	10,904,305	10,832,859	8,567,258	0.14523	0.79086
*E. coli*	BE	S	B	14,572,674	14,350,659	11,858,945	0.12384	0.82637
*E. coli*	AT	S	C	2,686,416	2,651,632	2,408,901	0.01921	0.90846
*E. coli*	AT	S	B	3,368,476	3,332,961	3,032,545	0.01958	0.90987
*E. coli*	AT	S	N	1,767,053	1,729,570	1,577,394	0.02130	0.91202
*E. coli*	AT	L	C	4,145,277	3,704,352	2,373,130	0.30522	0.64063
*E. coli*	AT	L	B	16,439,794	15,556,756	6,359,266	0.57286	0.40878
*E. h. onager*	BE	S	C	13,845,023	13,245,244	8,347,498	0.08795	0.63023
*E. h. onager*	BE	S	B	12,048,597	11,450,729	8,306,259	0.09053	0.72539
E. h. onager*	AT*	L*	C*	18,620,819*	17,631,778*	8,855,270*	0.15327*	0.50223*
*E. h. onager*	AT	L	B	2,855,334	2,656,430	1,765,944	0.14474	0.66478
*E. h. onager*	AT	L	N	3,594,737	3,322,966	2418284	0.07645	0.72775
*E. quagga quagga*	BE	S	NA	10,581,100	10,247,399	1,119,460	0.81341	0.10924
*E. quagga quagga*	AT	S	NA	62,447,876	26,768,728	15,673,713	0.14948	0.58552
*H. saldiasi*	BE	S	NA	13,556,850	12,937,410	20,490	0.10258	0.00158
*H. saldiasi*	AT	S	NA	8,371,861	5,720,486	8,424	0.30126	0.00147

The number of raw sequence read pairs generated as well as the number of collapsed trimmed reads and the number of unique hits to reference genomes and passing quality filters are indicated. Endogenous DNA content was calculated by dividing the total number of unique hits passing quality filters and the total number of collapsed reads. BE: Blunt-End adapter ligation. AT: AT-overhang adapter ligation. The final concentration of adapter used for ligation is reported as standard (S) or low (L; see Material and Methods). C: Covaris sonication. B: Bioruptor sonication. N: Nebulization. While most DNA libraries were sequenced as Paired-End (2×100 cycles), one, indicated with an asterisk, was sequenced as Single-End. For this DNA library, #Collapsed refers to the numbers of reads considered post-trimming and not post-collapsing, as for other DNA libraries.

### Sequence analysis

The overall sequence analysis procedure is based as previously described [21], [25], [34]. Briefly, prior to sequence alignment against modern reference genomes using BWA [35], each set of single-end or paired-end reads were processed using AdapterRemoval [36]; this involved removing the known adapter sequence, allowing a mismatch rate of 1/3, trimming low quality bases (Phred score 2 or base called as N) at read termini. We collapsed paired-end reads into single reads where the sequencing ends were found to overlap for at least 11 bp [3], [11]. Finally, processed reads shorter than 25 bp were discarded in order to limit noise resulting from misalignments. Following processing, reads were aligned to reference genomes (equids: EquCab2.0 autosomes; *E. coli*: Accession number NC_010473) using BWA v0.5.9-r26-dev, with ancient sequences aligned without a seed region as recommended in [34]. For ancient DNA, only paired-end reads collapsed into single reads were considered. Only high-quality hits (PHRED score ≥ 25) were retained. Finally, aligned single-end and paired-end reads for each sample were filtered for PCR duplicates using MarkDuplicates from the Picard toolkit [http://picard.sourceforge.net/], and using a script kindly provided by Martin Kircher (FilterUniqueBAM.py; for a SAM compatible version, see https://bioinf.eva.mpg.de/fastqProcessing/) for collapsed reads. *Post-mortem* damage patterns and DNA composition was estimated using mapDamage [24]. To estimate the fraction of PCR duplicates, high-quality hits *including* high-quality PCR duplicates were tabulated.

## Results

### Fresh DNA extracts

Fresh genomic extracts of *E. coli* were fragmented by sonication using a Covaris instrument. Identical aliquots of genomic extracts were further built into two standard types of Illumina DNA libraries, based on Blunt End or AT-overhang ligation (hereafter refered to as BE and AT libraries, respectively).

**Figure 1:**
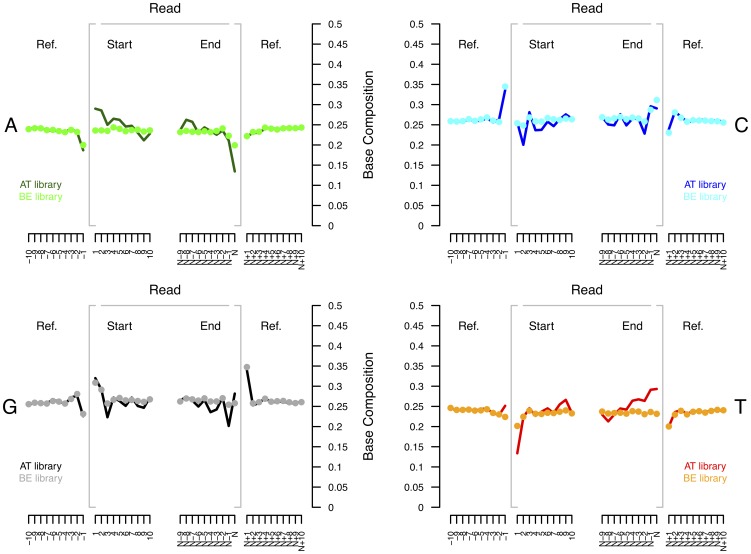
Base composition bias: AT *versus* BE libraries. Fresh aliquots of *E. coli* DNA extracts were sheared using the Covaris E210 sonicator, size selected, and built into AT and BE libraries (adapter concentration  =  0.6 µM). We report the base composition observed at the first 10 (positions 1 to 10) and last 10 (positions N-9 to N) nucleotide positions within sequence reads mapping with high quality a unique position of the *E. coli* NC_010473 genome. The genomic composition of the 10 nucleotides located upstream (positions –10 to –1) and downstream (positions N+1 to N+10) DNA inserts are also provided.

For BE libraries, the base composition was found to be homogenous across read positions, except for a slight deficit (4.5%) in thymine compared to the average base composition of the reference genome, and a slight enrichment (5.5%) in guanine contents at sequencing starts (Figure 1). However, the genomic position preceding sequencing starts appeared enriched (9.1%) in cytosine suggesting that genomic templates were slightly preferentially fragmented after cytosine residues and/or that the ligation step was slightly biased in favor of 3′-dC and 5′-dG termini. A complementary situation was observed at read ends where the last nucleotide position sequenced within reads was impoverished in adenine and enriched in cytosine while the first genomic location following sequencing reads was found to be enriched in guanine.

Strikingly, for AT libraries, base composition profiles showed strong deviation from the average genomic base composition within reads and a strong deficit (11.2% compared to the average base composition of the reference genome) in thymine residues at the start of sequences, that was paralleled by enrichment in adenine and guanine residues. Sequence read ends showed a complementary situation, with a strong deficit in adenine, and a slight increase in cytosine and thymine contents. The deficit (16.2%) of thymine at the start of sequences was even larger when AT libraries were constructed, with a low adapter concentration, on fresh *E. h. onager* genomic extracts (Figure S1). This was not observed for BE libraries. Such AT libraries also showed an increase (7.2%) in thymine at sequencing ends stronger than the one observed for libraries built on *E. coli* extracts.

This suggests that the molecular tools used for building Illumina DNA libraries affect the distribution of genomic inserts in a sequence-context dependent manner.

Overall, BE libraries show minimal base composition bias in contrast to AT libraries that introduce significant deviation to the expected base composition of the 10 nucleotide positions located at sequence read starts and ends. The latter has been proposed to result from the method used for shearing genomic templates [37], [38]. However, we found similar results with two other fragmentation methods (nebulization and Bioruptor; Figure S2), suggesting that the bias was introduced at the library building stage and not during fragmentation. Of note, the base compositions observed for DNA sheared using the Covaris and the Bioruptor sonicators were identical. The library built on nebulized DNA showed a similar profile, but with slightly higher adenine (1.27% compared to samples fragmented with Bioruptor) and thymine (1.26% compared to samples fragmented with Bioruptor) contents, paralleled by slightly lower cytosine and guanine compositions. Interestingly, the observation that the genomic position preceding sequencing starts was found to be enriched in cytosine residues for both BE and AT DNA libraries (Figure 1) indicates that DNA fragmentation preferentially cleaved DNA at 3' of cytosine residues. The fact that we amplified DNA libraries in emulsion suggests that the bias observed was not the result of potential competition among DNA templates during PCR amplification of DNA libraries. Of note, we found a similar deficit in thymine residues at sequence starts (coupled with an excess at sequencing ends) when repeating the same overall experimental approach, except that DNA libraries were amplified following a regular PCR protocol and not in emulsion (Figures S3, S4, S5, S6).

**Figure 2:**
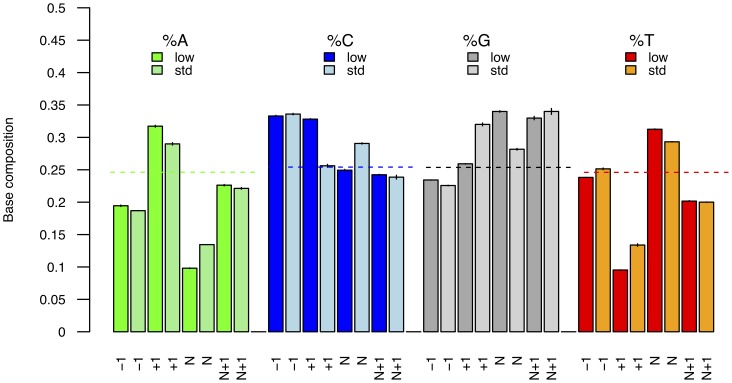
A low adapter concentration magnifies the base composition bias for AT libraries. Aliquots of *E. coli* DNA extracts were sheared using the Covaris E210 sonicator, size selected, and built into AT libraries using a low (“low”, 0.012 µM) or a standard (“std”, 0.6 µM) adapter concentration. We report the base composition observed at the start (position +1) and the end (position N) of sequences for reads mapping with high quality a unique position of the *E. coli* NC_010473 genome. The base compositions of the first nucleotide located upstream (position –1) and downstream (position N+1) read alignments are also provided. The average base composition is reported with dashed lines and is calculated using base counts within the reference genome.

We next addressed whether the adapter concentration used during library preparation could affect the population of inserts that are amenable to sequencing. AT libraries were built using *E. coli* genomic extracts fragmented with either Bioruptor or Covaris sonication and two concentrations of adapters (standard and low, see methods and Table 1). This test was limited to AT libraries due to the minimal bias observed in the base composition of sequencing reads generated from BE libraries. Interestingly, DNA libraries built with a higher adapter concentration showed reduced deviation in their base composition at sequencing starts, with 13.4% of thymine residues at the first nucleotide position sequenced in contrast to the expected average genomic composition (24.6%; Figure 2) and to the observed composition (9.5%) when using lower adapter concentration. Likewise, a lower adapter concentration decreased the level of adenine residues at sequencing ends.

Our findings principally have implications for genome sequencing where limited adapter concentrations are often used in order to limit the formation of spurious adapter dimers as this procedure magnifies the base composition bias and results in a limited access to DNA templates starting with thymine residues. We predicted this could affect our proficiency to ligate ancient DNA templates to adapters and therefore our ability to recover the full molecular complexity of ancient DNA extracts. Cytosine residues located at the ends of ancient DNA templates are indeed often deaminated into uracils [17], [18], which are molecular analogues to thymines. We next tested this prediction using ancient DNA templates extracted from a museum specimen and a Late Pleistocene extinct equid specimen.

### Ancient DNA extracts

**Figure 3:**
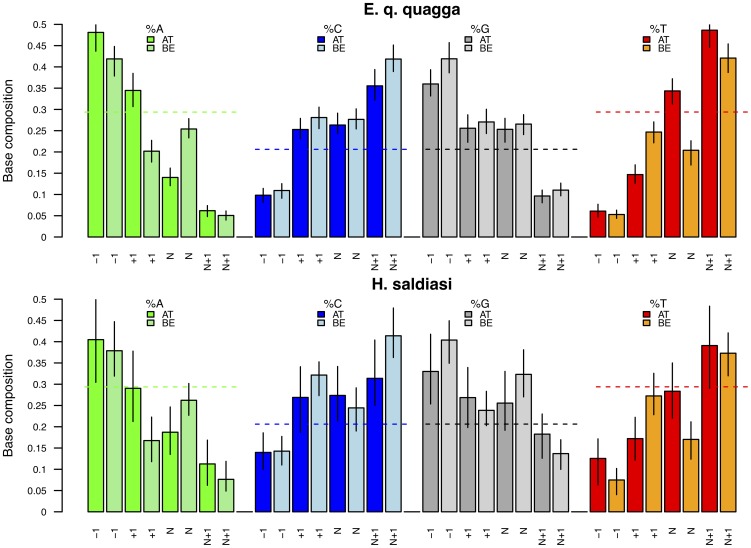
Base composition bias for ancient DNA templates: AT *versus* BE libraries. Aliquots of a quagga museum specimen and an *Hippidion* bone fossil were built into AT and BE libraries. See Figure 2 captions for further information regarding base compositions.

**Figure 4:**
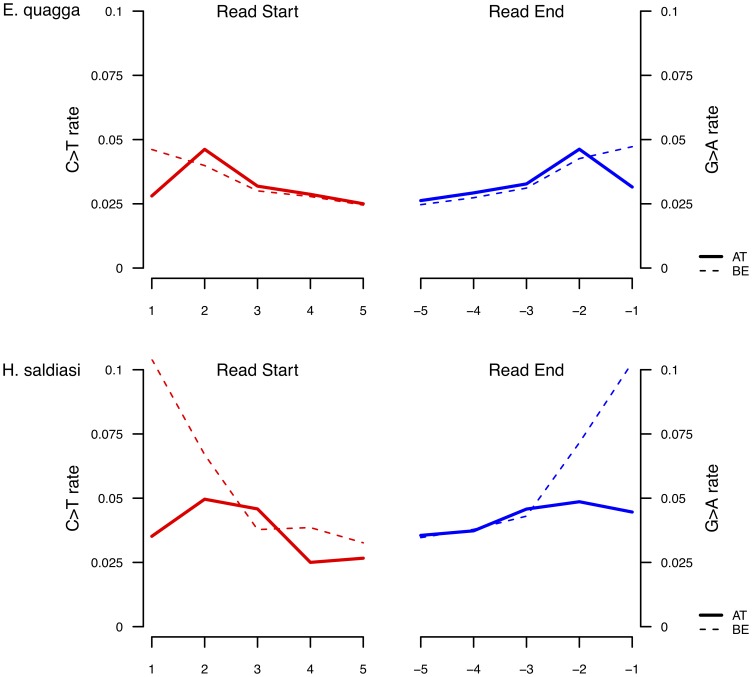
Nucleotide misincorporation bias for ancient DNA templates: AT *versus* BE libraries. Aliquots of a quagga museum specimen and an *Hippidion* bone fossil were built into AT and BE libraries. We report CT mismatch rates at the first 5 (positions 1 to 5) and last 5 (positions –1 to –5) nucleotide positions within sequence reads mapping with high quality a unique position of the EquCab2.0 genome. These rates are calculated using mapDamage output [24] by summing over positions where a C (G) is found in the reference genome but a T (A) is found in sequencing reads.

The museum specimen consisted of a quagga, which is an African equid species that became extinct in the wild at the end of the nineteenth century [39]. The Late Pleistocene equid sample belonged to the species *Hippidion saldiasi*, which populated Southern America from 2.5 million years ago until it became extinct some 10,000 years ago [33], [40]. Similar findings were observed on both specimens. Strikingly, for BE libraries, the base composition at the genomic position preceding (following) sequencing reads (ends) was found to be enriched in purines (pyrimidines), in agreement with previous reports [16], [20] (Figure 3). Additionally, misincorporation patterns at sequencing starts (C→T) and sequencing ends (G→A) were also in agreement with previous reports [16], [20], [21], [41] (Figure 4).

Importantly, when the same DNA extracts were built into AT libraries, the base composition of the genomic region preceding and following sequencing reads was also found to be enriched in purines and pyrimidines, respectively. Of note, among purines, adenine residues were found more enriched than guanine residues for the museum specimen as well as for the Late Pleistocene *Hippidion* (48.1% *vs* 36% for the quagga specimen; Figure 3). The complementary state was found at the genomic position located just after sequencing ends, with a greater rate of thymine than cytosine residues. This bias was also present, although less pronounced (40.5% *vs* 33%), for the *Hippidion* specimen. Of note, the same was observed using different cycling conditions for amplifying DNA libraries (Figure S7).

In addition, the frequency of thymine residues at the start of sequences was also found to be significantly reduced for AT libraries, but not BE libraries (14.7% *vs* 24.7% for the quagga specimen), a pattern consistent with the bias described above on modern DNA extracts. The strong bias against templates starting with thymine residues, which we found being specific to AT libraries, could limit our ability to ligate ancient DNA templates, especially those starting with deaminated cytosines (uracils). In agreement with our hypothesis, we observed for the quagga data, that C→T nucleotide misincorporation rates were inferior at read start positions (2.8%) than at the second nucleotide position (4.6%) (Figure 4). Similar patterns were observed using the Late Pleistocene *Hippidion* bone, even though AT libraries were gel purified following library amplification. Furthermore, the same was observed using different cycling conditions for amplifying DNA libraries (Figure S8), suggesting that the bias originated from the method used during library preparation (namely, the adapter ligation step) and not from the library amplification step. Bearing in mind what we observed from modern *E. coli* and *E. h. onager* extracts, where a higher adapter concentration reduced (but not removed) the extent of the bias, we could likely have limited this effect by using higher adapter concentration during library preparation. Our findings have important consequences for ancient and forensic DNA studies where both depurination and misincorporation patterns have been suggested as criteria for authenticating sequencing results generated from next-generation platforms [20].

## Discussion

In this study, we have demonstrated that DNA library preparation procedures based on AT-overhang adapter ligation are biased against DNA templates starting with thymine residues. This procedure is also affected by other sequence-dependent biases, as shown by reproducible patterns deviating from the average genomic base composition at sequencing read termini. In contrast to previous claims [37], [38], this bias is *not* a by-product of DNA fragmentation, but likely results from the ligation step. We found that reduced adapter concentrations, often used in order to reduce the formation of adapter dimers during library preparation, enhance the extent of the bias.

One important consequence of our finding is that different regions showing favourable sequence contexts will be over-represented following sequencing while other regions will be under-represented, resulting in uneven depth-of-coverage variations, error rates and consequently, data quality. This bias could admittedly be overcome by increasing sequencing efforts, something that becomes more feasible as sequencing costs decrease; however, our findings also reveal that heterogeneous datasets with non-optimal overlap will be obtained for low-coverage genomes especially when different library preparation procedures are used. This is of particular relevance for genomic projects involving limited amounts of DNA material, *e.g.* for non-invasive samples [42] or when genomes of single cells are targeted [43]. In addition to the bias described in this study, other sources of bias in Illumina sequencing have been reported and could impact the outcome of genomic surveys. Those range from the size selection step (with high-melting temperatures favouring %GC-rich regions; [29]), the amplification step (with a full range of possible size-dependent and %GC-dependent biases depending on which *Taq* DNA polymerase is used [44]), to artifacts in base calling and image analysis [45], [46].

That AT-overhang ligation is biased against DNA templates with 5′-dT has major consequences for ancient DNA research and museomics. We know now that most ancient DNA templates contain overhanging ends [4], [16], [21]. At such sites, cytosine residues show increased rates of deamination into uracils [17], [18], a chemical analogue to thymines. As a result, the incorporation of deaminated ancient DNA templates into DNA libraries could be sub-optimal, especially compared to non-deaminated fresh DNA contaminants. This in turn could reduce the ability of the AT-overhang ligation preparation procedure to access the full fraction of endogenous DNA that is preserved in the extract, and consequently could reduce the molecular complexity of ancient DNA inserts. In situations where the prominent fraction of exogenous DNA molecules originates from environmental microbes with AT-rich genomes, this could provide an advantage to AT libraries and result in endogenous DNA content greater than what is observed with BE libraries. Conversely, with balanced base compositions between microbial and endogenous DNA fractions, the bias described here could result in endogenous DNA contents that are lower for AT libraries than for BE libraries. Given that ancient DNA extracts show variable microbial compositions [3], [6], how much the detected bias could affect the expected endogenous DNA content will likely be difficult to predict, and could potentially explain why slightly higher endogenous contents were gathered using BE libraries for *Hippidion* extracts (0.158% *vs* 0.147%) while AT libraries performed better for the quagga (58.6% *vs* 10.9%).

One consequence of the detected bias related to ligation at AT-overhangs was found to be more predictable. We found that the level of C→T misincorporations observed at sequencing starts (G→A at sequencing ends) was reduced for AT libraries compared to BE libraries. This results from the fact that templates showing deaminated cytosines at 5′-ends have lower chances to be ligated to adapters than templates showing no deamination, resulting in reduced deamination-driven misincorporation rates at those positions. This generally results in peaks of C→T and G→A misincorporation rates at the second position and the second to the last position of sequencing reads, respectively (although peaks shifted by one-additional offset have been observed in some cases; L. Orlando, unpublished results). Due to lower-than-expected misincorporation rates at sequencing starts and ends, ancient DNA libraries prepared following the AT-overhang ligation procedure will systematically provide an under-estimation of the overall DNA damage levels compared to those prepared with the BE ligation procedure. This implies that quantitative comparative analyses of DNA damage levels across samples coming from temporal and/or environmental gradients must be precluded unless *(i)* sequence datasets have been generated using identical types of library preparation procedure or *(ii)* adequate DNA damage models, capturing the specificities of the molecular tools used during library preparation, are used. Such analyses will be essential for estimating empirical rates of DNA decay after death *in situ* in different environmental and/or sample types (e.g. bone [3], [4], teeth [8], hair [1], [2], coprolites [47], eggshells [48]). Statistical models of DNA damage have recently been released [16], [27]. Among those, mapDamage 2.0 does not require a geometric decrease (increase) in C→T (G→A) misincorporations at sequencing starts (ends), which makes it fully versatile to the molecular tools used for building DNA libraries. With such models, DNA damage rates can potentially be explored over a full range of taphonomical contexts and using the data gathered from a full variety of experimental procedures. Such analyses will advance our understanding of DNA decay after death [49] and will complement current empirical estimates that are only commencing to become available [22], [30], [50].

Analysis of fresh modern DNA also revealed that the genomic position preceding sequencing starts is more often than expected a cytosine residue. This was true for both AT-overhang and BE ligation protocols and for all different fragmentation procedures investigated in our study. This likely reflects a systematic bias in DNA fragmentation 3′ of such residues. This finding has important consequences for ancient DNA research where excess of the purines adenine and guanine are generally observed at such genomic coordinates. The latter has been shown for both library preparation procedures investigated in this study, but also using single-strand based approaches, followed either by Illumina sequencing [4] or Helicos sequencing [21], and over a wide diversity of samples originating from full range of time periods and/or preservation environments [3], [18], [51]. This suggests that different fragmentation mechanisms are at play for fresh modern DNA templates and ancient DNA (where depurination mainly drives fragmentation *post-mortem* [16]), validating the use of an excess of purines at this position as an important authentication criterion.

## Conclusion

We have shown that library preparation procedures based on AT-overhang adapter ligation introduce significant bias in the base composition of Illumina libraries. Together with variations in mappability across regions, this bias results in an uneven representation of different genomic landscapes. This has important consequences in structuring the lateral coverage of genome datasets sequenced at low depth and should be considered upfront in any project involving single cell genome sequencing, non-invasive samples, museomics and ancient DNA where DNA material is scarce. It also significantly limits our ability to successfully integrate into libraries the whole molecular complexity of DNA templates preserved in ancient extracts. Finally, it substantially changes the shape of nucleotide misincorporation patterns that are often used as important authentication criteria in ancient DNA research, leading to a systematic under-estimation of the levels of *post-mortem* cytosine deamination. Characterizing which nucleotide misincorporation patterns are expected for different types of library preparation methods (and can be used as signatures of *post-mortem* damage) is an important step forward as it opens the possibility of filtering sequence reads deviating from the expected pattern and therefore remove most contamination sources.

## Supporting Information

Figure S1 **Base composition bias for onager templates: AT** ***versus*** **BE libraries.** Fresh aliquots of *E. h. onager* DNA extracts were sheared using the Covaris E210 sonicator, size selected, and built into AT libraries (adapter concentration  =  0.012 µM) or BE libraries (adapter concentration  =  0.6 µM). See Figure 1 captions for further information regarding base compositions.
(TIFF)

Figure S2 **Effect of the DNA fragmentation method on the base composition bias for AT libraries.** Fresh aliquots of *E. coli* DNA extracts were sheared using the Covaris E210 sonicator, the Bioruptor or nebulizers. The fragmented DNA was size selected and built into AT libraries (adapter concentration  =  0.6 µM). See Figure 1 captions for further information regarding base compositions.
(TIFF)

Figure S3 **Base composition bias: AT** ***versus*** **BE libraries built on bacterial DNA sheared using the Bioruptor.** Fresh aliquots of *E. coli* DNA extracts were sheared using the Bioruptor sonicator, size selected, and built into AT or BE libraries (adapter concentration  =  0.6 µM). The libraries were amplified by regular PCR, not in emulsion. See Figure 1 captions for further information regarding base compositions.
(TIFF)

Figure S4 **Base composition bias: AT** ***versus*** **BE libraries built on bacterial DNA sheared using the Covaris sonicator.** Fresh aliquots of *E. coli* DNA extracts were sheared using the Covaris sonicator, size selected, and built into AT or BE libraries (adapter concentration  =  0.6 µM). The libraries were amplified by regular PCR, not in emulsion. See Figure 1 captions for further information regarding base compositions.
(TIFF)

Figure S5 **Base composition bias: AT libraries built on DNA sheared using Covaris** ***versus*** **Bioruptor sonicators.** Fresh aliquots of *E. h. onager* DNA extracts were sheared using the Bioruptor or the Covaris sonicator, size selected, and built into AT libraries (adapter concentration  =  0.012 µM). See Figure 1 captions for further information regarding base compositions.
(TIFF)

Figure S6 **Effect of the fragmentation method on the base composition bias for AT libraries.** Fresh aliquots of *E. coli* DNA extracts were sheared using the Covaris E210 sonicator, the Bioruptor or nebulizers. The fragmented DNA was size selected and built into AT libraries (adapter concentration  =  0.6 µM). The libraries were amplified by regular PCR, not in emulsion. See Figure 1 captions for further information regarding base compositions.
(TIFF)

Figure S7 **Base composition bias for ancient DNA templates: AT** ***versus*** **BE libraries amplified using a short elongation step.** Aliquots of a quagga museum specimen and an *Hippidion* bone fossil were built into AT or BE libraries, and amplified with PCR conditions using a short (40 sec) elongation step. See Figure 2 captions for further information regarding base compositions.
(TIFF)

Figure S8 **Nucleotide misincorporation bias for ancient DNA templates: AT** ***versus*** **BE libraries amplified using a short elongation step.** Aliquots of an *Hippidion* bone fossil were built into AT or BE libraries, and amplified with PCR conditions using a short (40 sec) elongation step. See Figure 4 captions for further information regarding base compositions.
(TIFF)

Material and Methods S1 **Modern DNA library amplification in absence of emulsion.**
(DOCX)

Material and Methods S2 **Ancient DNA library amplification using a short elongation step.**
(DOCX)
