# Supplementary figures and images for: Ligation Bias in Illumina Next-Generation DNA Libraries: Implications for Sequencing Ancient Genomes
Source: PLoS One. 2013 Oct 29;8(10):e78575. doi: 10.1371/journal.pone.0078575 (PMC3812280; doi:10.1371/journal.pone.0078575)

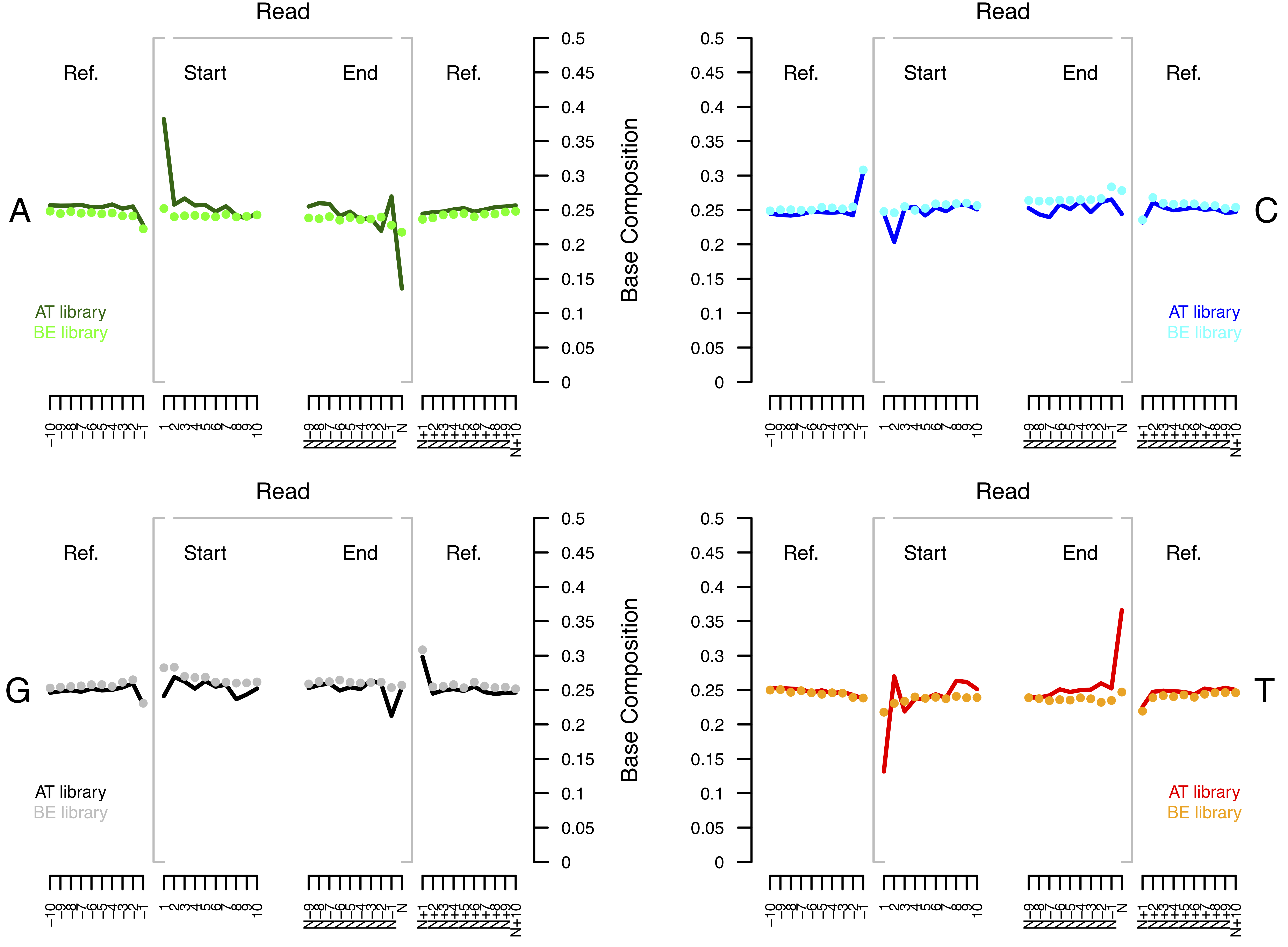

Supplement: Figure S1 — Base composition bias for onager templates: AT versus BE libraries. Fresh aliquots of E. h. onager DNA extracts were sheared using the Covaris E210 sonicator, size selected, and built into AT libraries (adapter concentration = 0.012 µM) or BE libraries (adapter concentration = 0.6 µM). See Figure 1 captions for further information regarding base compositions. (TIFF) [file pone.0078575.s001.tif]

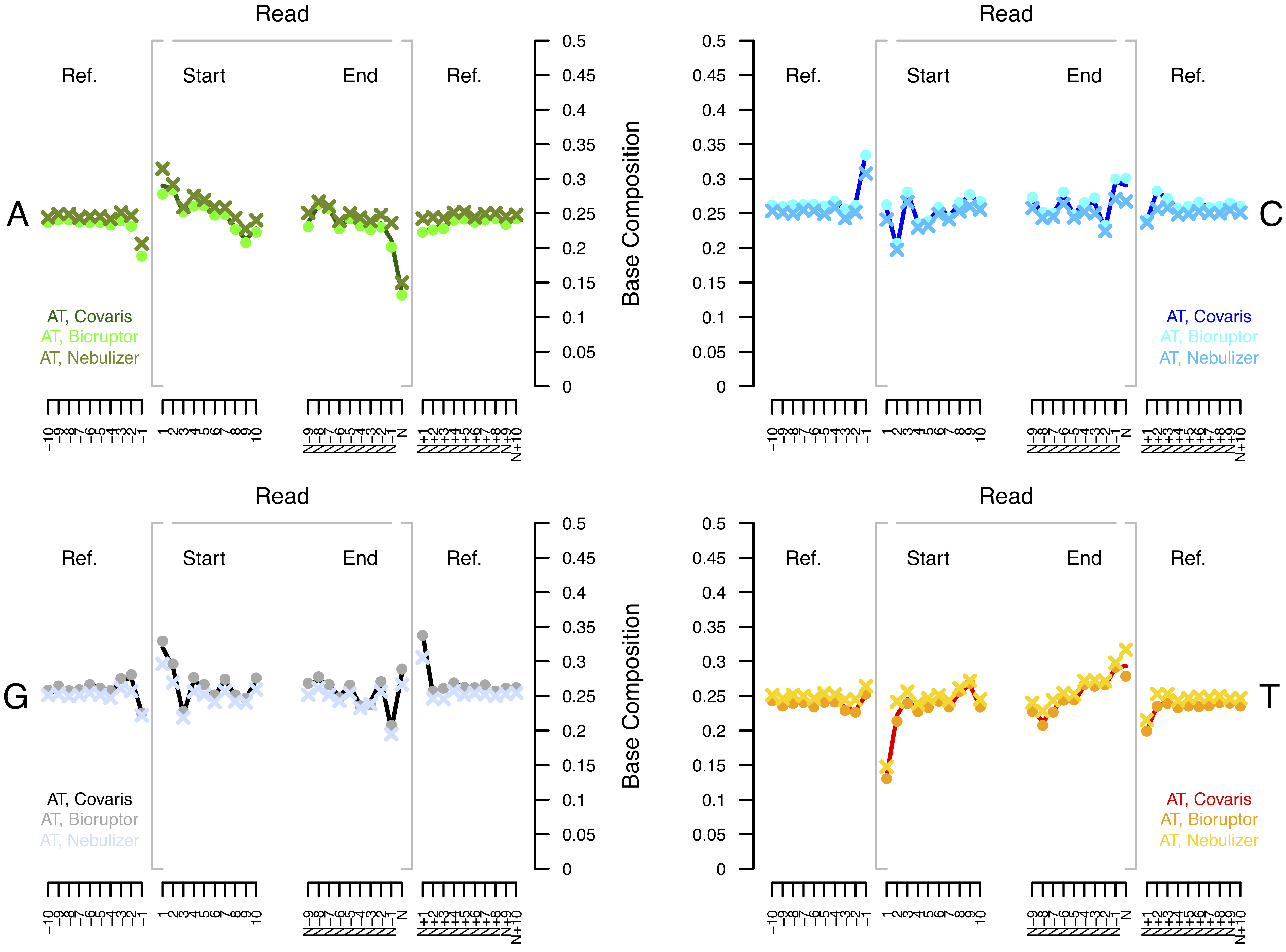

Supplement: Figure S2 — Effect of the DNA fragmentation method on the base composition bias for AT libraries. Fresh aliquots of E. coli DNA extracts were sheared using the Covaris E210 sonicator, the Bioruptor or nebulizers. The fragmented DNA was size selected and built into AT libraries (adapter concentration = 0.6 µM). See Figure 1 captions for further information regarding base compositions. (TIFF) [file pone.0078575.s002.tif]

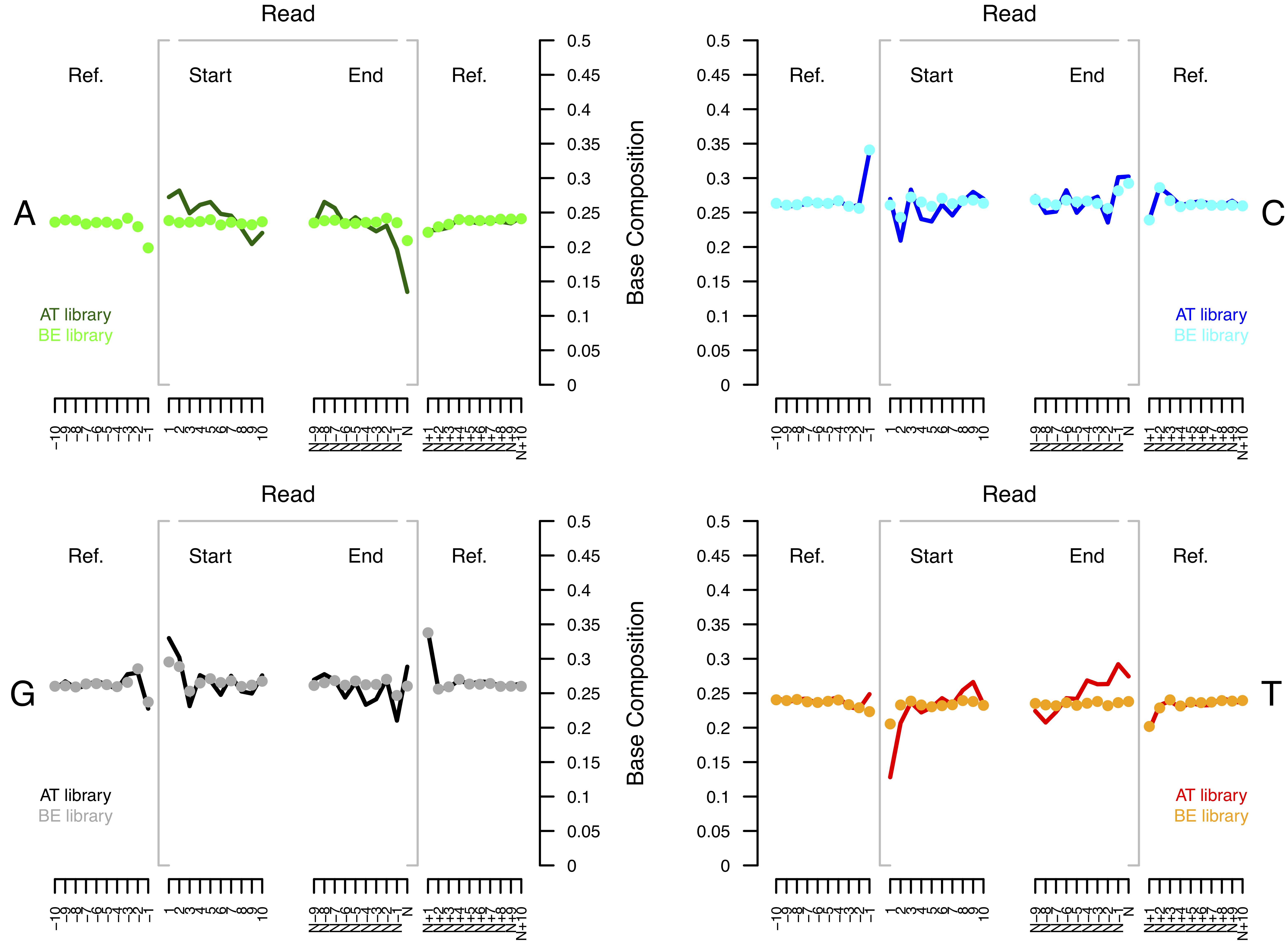

Supplement: Figure S3 — Base composition bias: AT versus BE libraries built on bacterial DNA sheared using the Bioruptor. Fresh aliquots of E. coli DNA extracts were sheared using the Bioruptor sonicator, size selected, and built into AT or BE libraries (adapter concentration = 0.6 µM). The libraries were amplified by regular PCR, not in emulsion. See Figure 1 captions for further information regarding base compositions. (TIFF) [file pone.0078575.s003.tif]

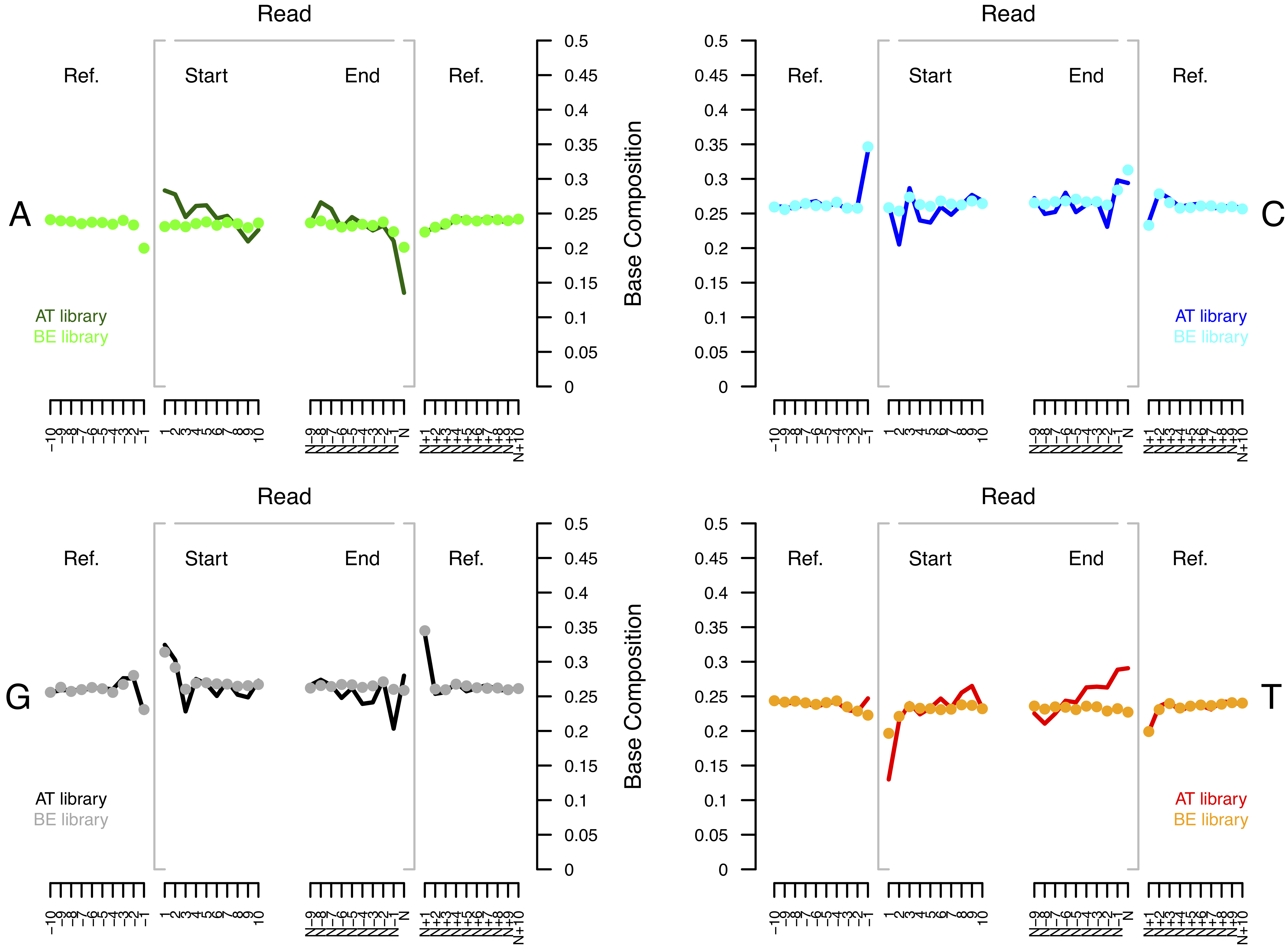

Supplement: Figure S4 — Base composition bias: AT versus BE libraries built on bacterial DNA sheared using the Covaris sonicator. Fresh aliquots of E. coli DNA extracts were sheared using the Covaris sonicator, size selected, and built into AT or BE libraries (adapter concentration = 0.6 µM). The libraries were amplified by regular PCR, not in emulsion. See Figure 1 captions for further information regarding base compositions. (TIFF) [file pone.0078575.s004.tif]

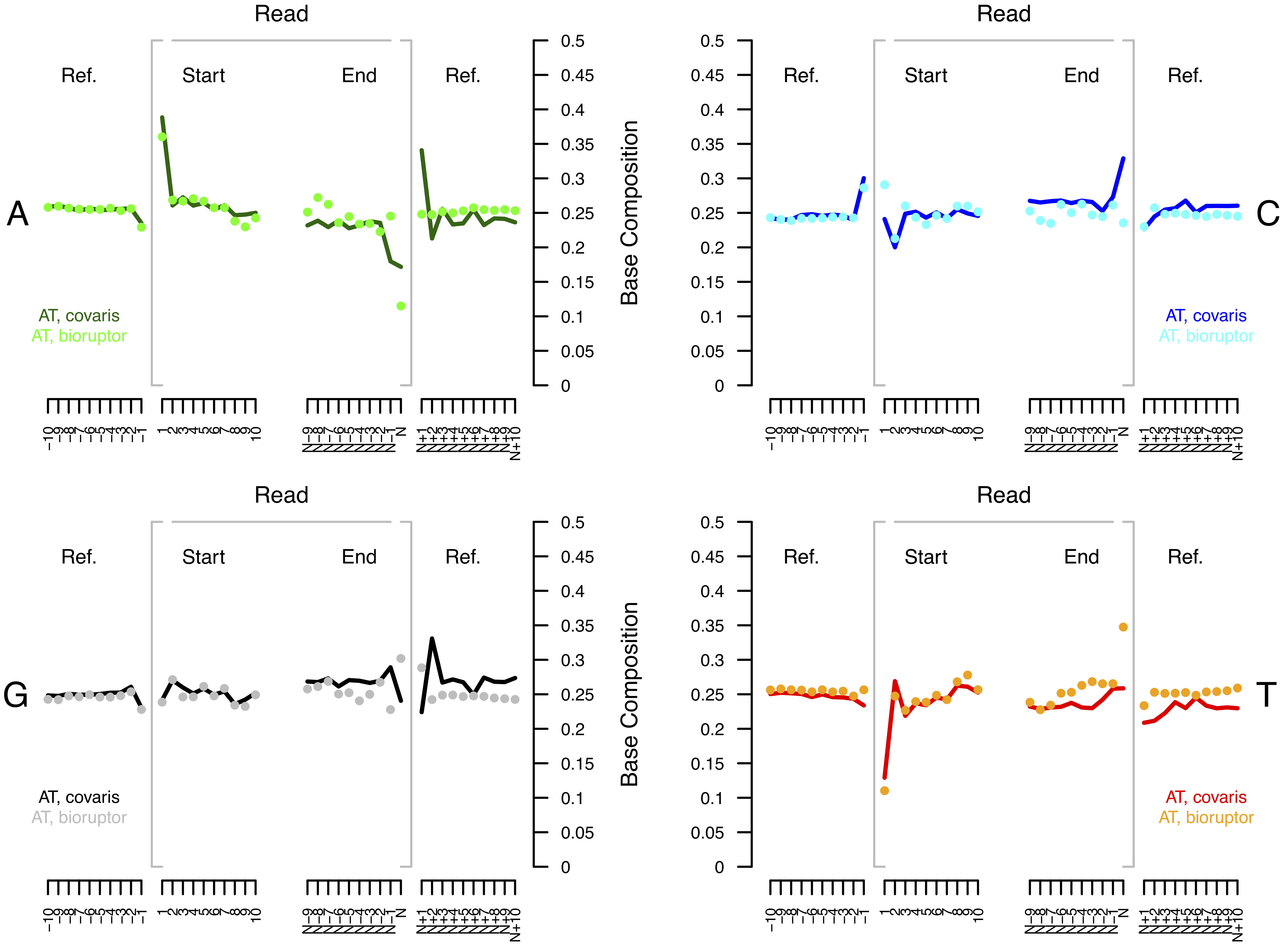

Supplement: Figure S5 — Base composition bias: AT libraries built on DNA sheared using Covaris versus Bioruptor sonicators. Fresh aliquots of E. h. onager DNA extracts were sheared using the Bioruptor or the Covaris sonicator, size selected, and built into AT libraries (adapter concentration = 0.012 µM). See Figure 1 captions for further information regarding base compositions. (TIFF) [file pone.0078575.s005.tif]

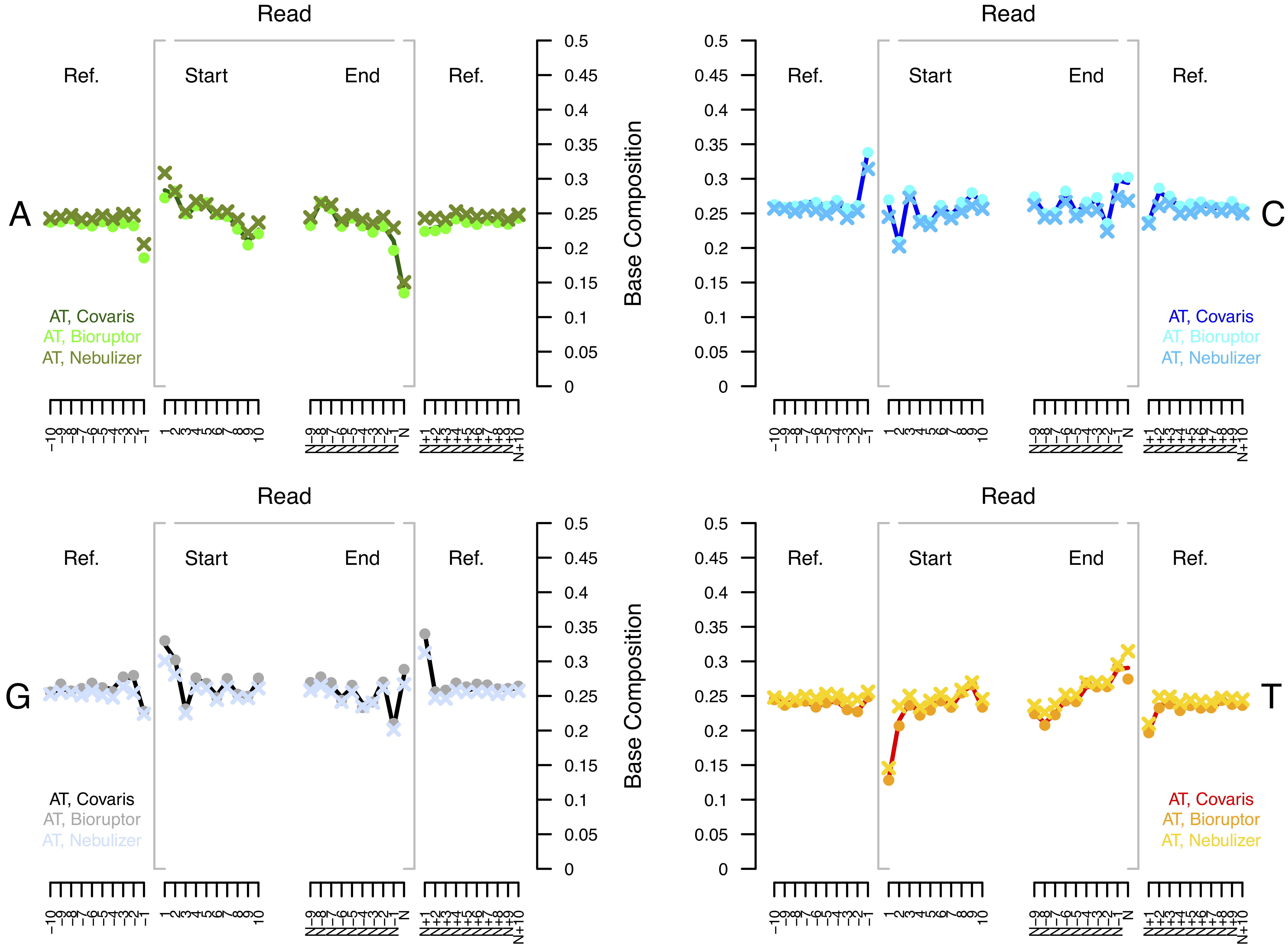

Supplement: Figure S6 — Effect of the fragmentation method on the base composition bias for AT libraries. Fresh aliquots of E. coli DNA extracts were sheared using the Covaris E210 sonicator, the Bioruptor or nebulizers. The fragmented DNA was size selected and built into AT libraries (adapter concentration = 0.6 µM). The libraries were amplified by regular PCR, not in emulsion. See Figure 1 captions for further information regarding base compositions. (TIFF) [file pone.0078575.s006.tif]

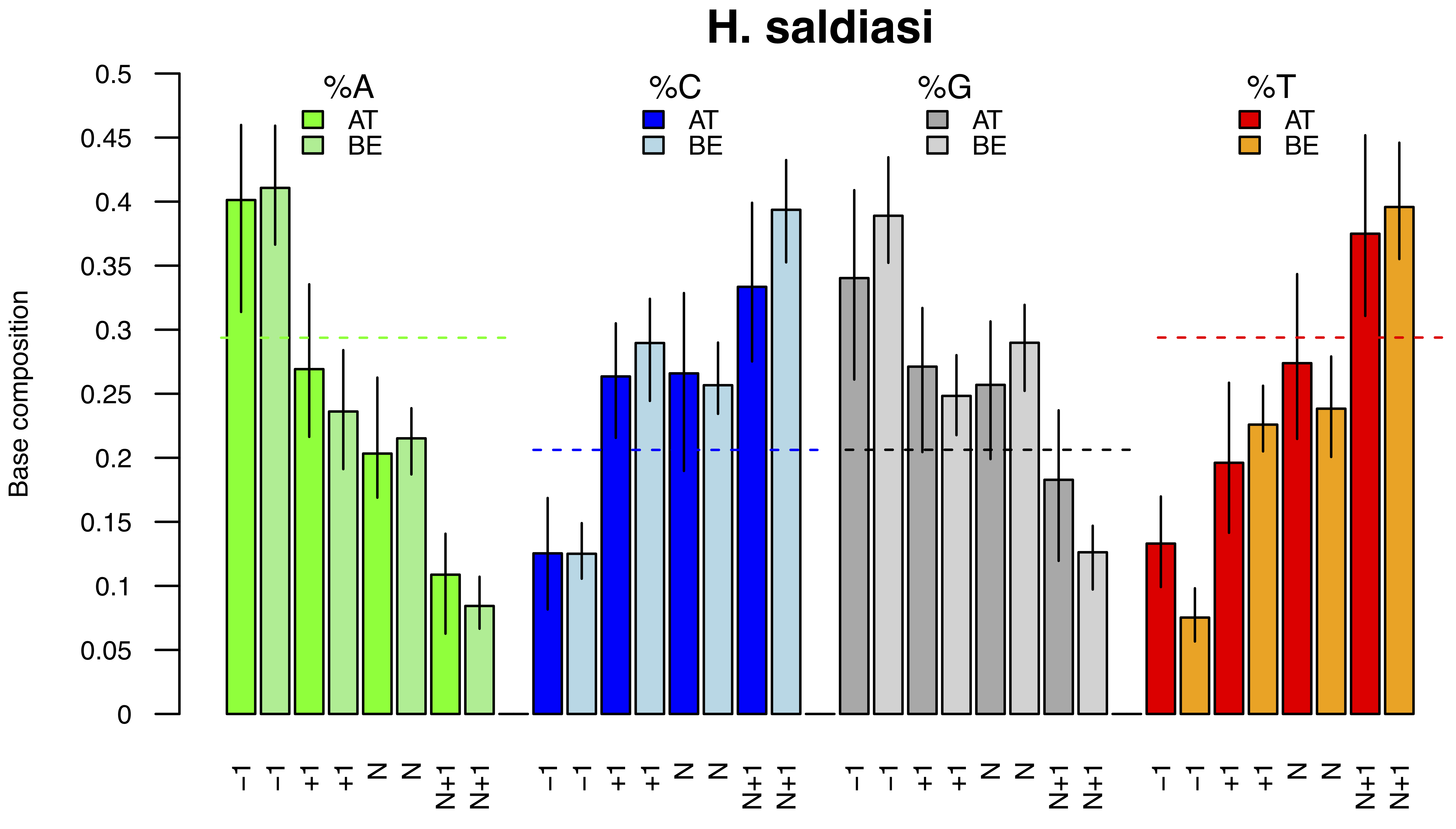

Supplement: Figure S7 — Base composition bias for ancient DNA templates: AT versus BE libraries amplified using a short elongation step. Aliquots of a quagga museum specimen and an Hippidion bone fossil were built into AT or BE libraries, and amplified with PCR conditions using a short (40 sec) elongation step. See Figure 2 captions for further information regarding base compositions. (TIFF) [file pone.0078575.s007.tif]

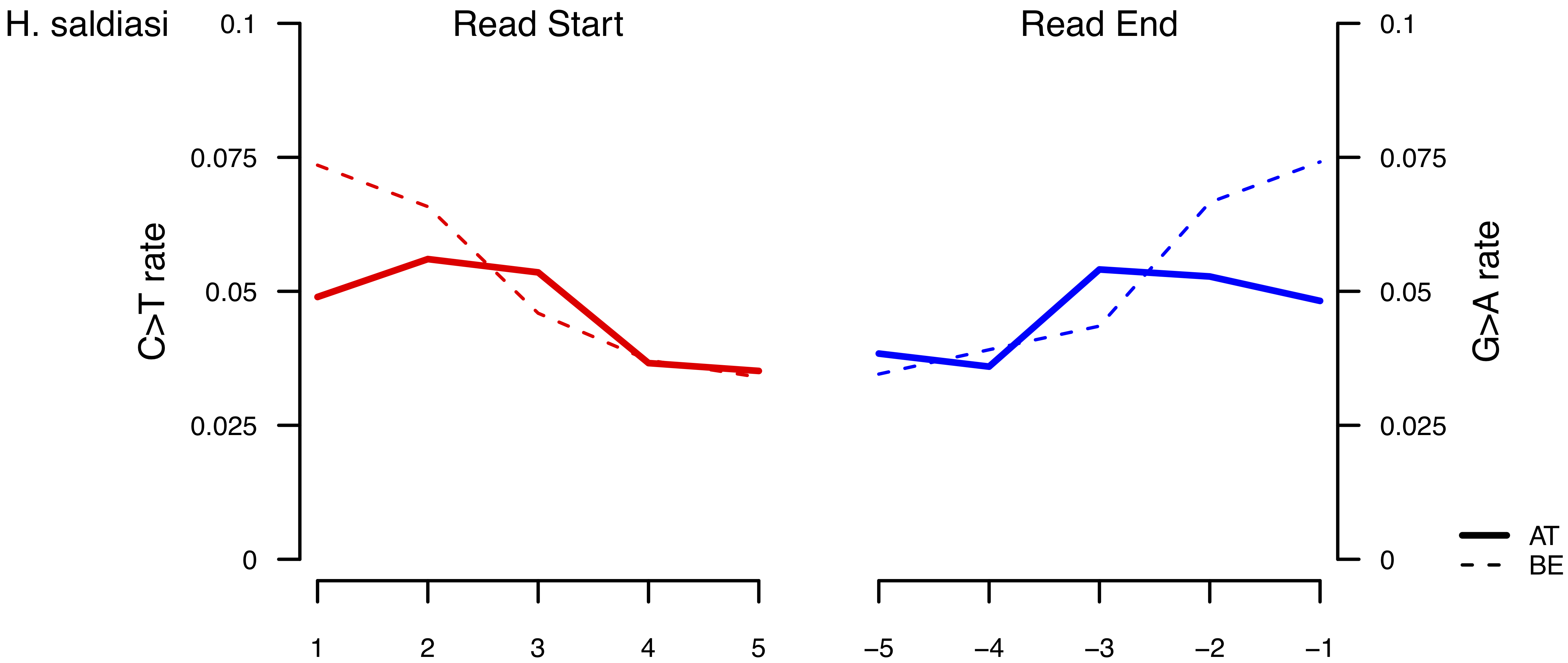

Supplement: Figure S8 — Nucleotide misincorporation bias for ancient DNA templates: AT versus BE libraries amplified using a short elongation step. Aliquots of an Hippidion bone fossil were built into AT or BE libraries, and amplified with PCR conditions using a short (40 sec) elongation step. See Figure 4 captions for further information regarding base compositions. (TIFF) [file pone.0078575.s008.tif]
