# Supplementary material for: Ligation Bias in Illumina Next-Generation DNA Libraries: Implications for Sequencing Ancient Genomes
Source: PLoS One. 2013 Oct 29;8(10):e78575. doi: 10.1371/journal.pone.0078575 (PMC3812280; doi:10.1371/journal.pone.0078575)
Supplement: Material and Methods S1 — Modern DNA library amplification in absence of emulsion. (DOCX) [file pone.0078575.s009.docx]

**Material and Methods S1: Modern DNA library amplification in absence of emulsion.**

DNA libraries prepared on fresh DNA extracts were also amplified in absence of emulsion. The reaction consisted of a 50 µl volume PCR mix, using 5 µl of DNA library, 1 µM of Primer inPE1.0 (5’-AAT GAT ACG GCG ACC ACC GAG ATC TAC ACT CTT TCC CTA CAC GAC GCT CTT CCG ATC T), 20 nM of primer inPE2.0 (5’-GTG ACT GGA GTT CAG ACG TGT GCT CTT CCG ATC T), 1 µM of an Illumina multiplex primer (5’-CAA GCA GAA GAC GGC ATA CGA GAT NNN NNN GTG ACT GGA GTT C, where the N stretch corresponds to a 6 nucleotide index tag), 25 µl *Phusion* High-Fidelity PCR Master Mix (NEB, ref : M0532) and nuclease free water qsp 50 µl.

PCR cycling conditions consisted of initial denaturation for 30 sec at 98°C, followed by 15 cycles of 10 sec denaturation at 98°C, 30 sec annealing at 65°C and 30 sec elongation at 72°C. Lastly, there was a final 5 min elongation step at 72°C. PCR products were purified on MinElute columns and eluted in 20 µl EB following 15 min incubation at 37°C.
