# Supplementary material for: Ligation Bias in Illumina Next-Generation DNA Libraries: Implications for Sequencing Ancient Genomes
Source: PLoS One. 2013 Oct 29;8(10):e78575. doi: 10.1371/journal.pone.0078575 (PMC3812280; doi:10.1371/journal.pone.0078575)
Supplement: Material and Methods S2 — Ancient DNA library amplification using a short elongation step. (DOCX) [file pone.0078575.s010.docx]

**Material and Methods S2: Ancient DNA library amplification using a short elongation step.**

The libraries prepared on ancient extracts were also amplified with shorter elongation steps. The libraries were first amplified in a 50 µl volume reaction using 5 µl of DNA library, and 5 units *Taq* Gold (Life Technologies), 1X Gold Buffer, 4 mM MgCl2, 1 mg/ml BSA, 62.5 µM of each dNTP, 0.5 µM of Primer inPE1.0, 10 nM of Primer inPE2.0 and 0.5 µM of an Illumina multiplex primer, as described for the modern samples. PCR cycling conditions consisted of initial denaturation for 10 min at 92°C, followed by 12 cycles of 30 sec denaturation at 92°C, 30 sec annealing at 65°C and 40 sec elongation at 72°C. Lastly, there was a final 7 min elongation step at 72°C. PCR products were purified on a MinElute column and eluted in 20 µL EB following 15 min incubation at 37°C. A second round of PCR amplification was then performed by splitting the purified product of the first PCR amplification into four reactions of 50 µl each using similar conditions but without inPE2.0 primer and only using 10 cycles. The four reactions were pooled and purified on a single MinElute column, eluted in 20 µl EB following 15 min incubation at 37°C.
